# Interaction of Hoechst 33342 with POPC Membranes at Different pH Values

**DOI:** 10.3390/molecules28155640

**Published:** 2023-07-25

**Authors:** Margarida M. Cordeiro, Hugo A. L. Filipe, Patrícia dos Santos, Jaime Samelo, João P. Prates Ramalho, Luís M. S. Loura, Maria J. Moreno

**Affiliations:** 1 Coimbra Chemistry Center, Institute of Molecular Sciences (CQC-IMS), University of Coimbra, 3004-535 Coimbra, Portugal; mmc.margarida0@gmail.com (M.M.C.); hlfilipe@ipg.pt (H.A.L.F.); jsamelo@uc.pt (J.S.); 2 Department of Chemistry, Faculty of Sciences and Technology, University of Coimbra, 3004-535 Coimbra, Portugal; 3 Polytechnic of Guarda, CPIRN-IPG—Center of Potential and Innovation of Natural Resources, 6300-559 Guarda, Portugal; 4 LAQV, REQUIMTE, Hercules Laboratory, Department of Chemistry, School of Science and Technology, University of Évora, 7000-671 Évora, Portugal; jpcar@uevora.pt; 5 CNC—Center for Neuroscience and Cell Biology, University of Coimbra, 3004-535 Coimbra, Portugal; 6 Faculty of Pharmacy, University of Coimbra, 3000-548 Coimbra, Portugal

**Keywords:** biomembranes, cationic amphiphiles, fluorescent probe, membrane partition, molecular dynamics simulations, POPC

## Abstract

Hoechst 33342 (H33342) is a fluorescent probe that is commonly used to stain the DNA of living cells. To do so, it needs to interact with and permeate through cell membranes, despite its high overall charge at physiological pH values. In this work, we address the effect of pH in the association of H33342 with lipid bilayers using a combined experimental and computational approach. The partition of H33342 to 1-palmitoyl-2-oleoyl-*sn*-glycero-3-phosphocholine (POPC) lipid membranes was experimentally quantified using fluorescence spectroscopy and isothermal titration calorimetry (ITC) measurements. Quantum chemical calculations were performed to select the most stable isomer of H33342 for the overall charges 0, +1, and +2, expected to predominate across the 5 < pH < 10 range. The interaction of these isomers with POPC bilayers was then studied by both unrestrained and umbrella sampling molecular dynamics (MD) simulations. Both experimental results and computational free energy profiles indicate that the partition coefficient of H33342 displays a small variation over a wide pH range, not exceeding one order of magnitude. The enthalpy variation upon partition to the membrane suggests efficient hydrogen bonding between the probe and the lipid, namely, for the protonated +2 form, which was confirmed in the MD simulation studies. The relatively high lipophilicity obtained for the charged species contrasts with the decrease in their general hydrophobicity as estimated from octanol/water partition. This highlights the distinction between lipophilicity and hydrophobicity, as well as the importance of considering the association with lipid bilayers when predicting the affinity for biomembranes.

## 1. Introduction

Hoechst 33342 (H33342) is a bis-benzimidazole derivative fluorescent dye that binds to the minor grove of B-DNA [1]. As is the case with many hydrophobic dyes that associate with DNA at the minor or major groves, the decrease in hydration and increase in the rigidity of the environment leads to a strong increase in the fluorescence quantum yield [2,3,4,5], allowing for the visualization of cell nuclei by optical microscopy. Hoechst 33342 permeates efficiently through cell membranes and can be used to stain the DNA of living cells [6,7]. Although H33342 transport has been reported to occur by active influx [8] and/or efflux [8,9,10] depending on the cells, a relatively fast passive permeation has also been shown, which allows for equilibration through the membrane to occur within a few minutes [6]. The specificity of H33342 to DNA and its fast permeation have recently extended the use of this popular probe as a targeting moiety in drugs directed towards cell DNA [11]. The high permeability of H33342 is quite unexpected, because at physiological pH, it is expected to have an overall positive charge [12].

In addition to its applicability in DNA staining and targeting, H33342 transport by P-glycoprotein has been extensively used in competition assays to characterize the transport of drugs by this efflux transporter [13,14]. To be transported by P-glycoprotein, the substrates must access the binding pocket from the inner face of the cell membrane [15]. Therefore, molecules added to the medium outside the cell must first associate with the membrane’s outer leaflet and permeate the lipid bilayer towards the inner leaflet. These processes will be affected by the ionization state of the protein substrates, altering the overall efflux transport. To understand the details in the protein activity, it is therefore important to know how the ionization state of H33342 as a substrate for P-glycoprotein affects its interaction with lipid membranes. 

The characterization of the effect of H33342 ionization on its interaction with lipid membranes extends well beyond this fluorescent probe and its use in staining DNA or characterizing P-gp’s activity. In fact, the knowledge obtained may be of relevance for drugs containing the imidazole and benzimidazole groups, some of which are already present in the market [16,17,18]. The prevalence of heterocyclic scaffolds in the drug chemical space is due to several physico-chemical properties that facilitate their interaction with the specific target and lead to good pharmacokinetics properties. Of particular relevance is their low conformational flexibility, leading to a low entropy penalty upon binding to macromolecules or membranes [19]; their moderate polarity, leading to a relatively high lipophilicity while maintaining a good solubility in aqueous media; and the electronic resonance that decreases the basicity of the nitrogen atoms, leading to a relatively high abundance of uncharged species at neutral pH and to the delocalization of the charge, thus decreasing the Born energy and facilitating their permeation through biomembranes [20,21,22].

**Figure 1:**
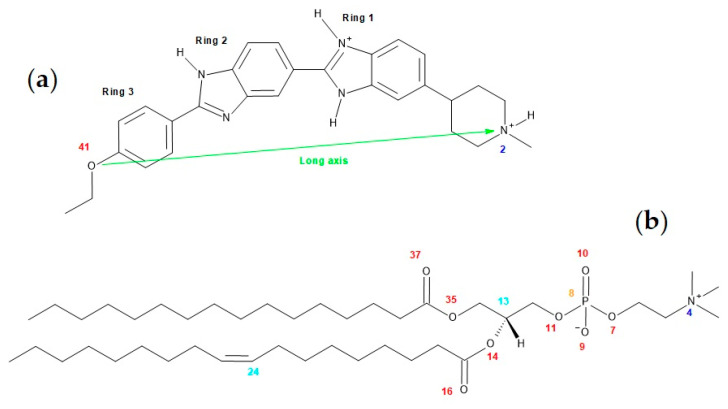
(**a**) Structures of one of the isomers of H33442 with overall charge +2. (**b**) Structure of POPC. In both structures, numbers of selected atoms (relevant for the MD analyses) are indicated. In panel (**a**), the H33342 long axis is also defined.

**Figure A1:**
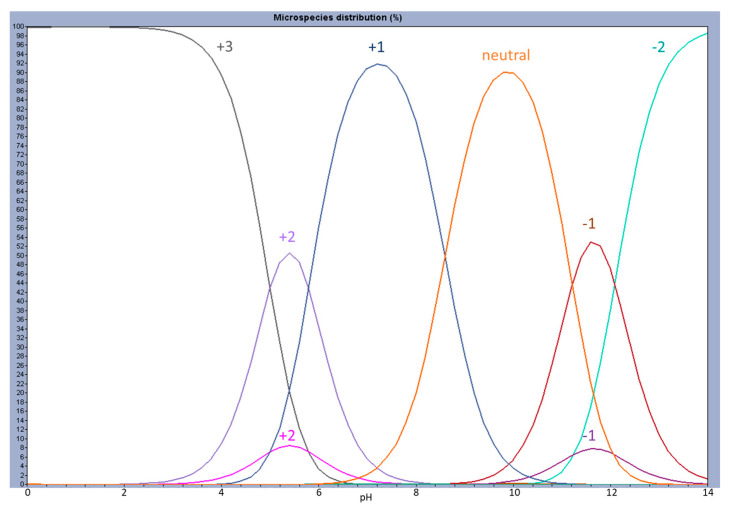
H33342 microspecies distribution (%) as a function of pH, adapted from the figure generated by the MarvinSketch software. The global charge of the most abundant species is indicated near the corresponding distribution curve.

Figure 1a shows the molecular structure of H33342, highlighting the three ionizable groups, two benzimidazole groups in the central region, and an aliphatic tertiary amine in one of the ends of the molecule. Usually, the aliphatic tertiary amines are relatively strong bases and are protonated at physiological pH (≈7). Quantum mechanical calculations carried out for H33342 indicated that one of the benzimidazole groups shows higher basicity than the other, with a pKa difference of two units [12]. The attribution given for the ionization constants in that work was made based on the acid/base properties of imidazole, resulting in pKa values for H33342 on the order of 5 and 7, or 7 and 9, dependent upon which group is considered to have an acid/base behavior similar to the reference imidazole base. Therefore, a global charge between +1.5 and +2.5 is predicted for H33342 at neutral pH, with a large variation within the range of physiological pH values [23]. The distribution of the different microspecies was calculated using the MarvinSketch software version 22.9.0 [24] and is shown in Appendix A (Figure A1) for the full pH range.

In this work, we characterize the interaction of H33342 with lipid membranes of 1-palmitoyl-2-oleoyl-*sn*-glycero-3-phosphocholine (POPC; structure in Figure 1b) in the pH range between 3 and 11. This lipid was chosen due to the high abundance of phosphatidylcholines in biological membranes [25,26,27,28,29] and the prevalence of the combination of palmitoyl and oleoyl acyl chains in the membrane phospholipids [30]. The H33342 partition coefficient (*K*_P_) was obtained from changes in its fluorescence intensity and fluorescence anisotropy. Isothermal titration calorimetry (ITC) was also used to characterize the partition coefficient, from the heat evolved in the interaction between H33342 and the lipids in the bilayer. In addition to the partition coefficient, with this methodology, the enthalpy variation (Δ*H*^o^) associated with the interaction is also obtained, providing important insight regarding the nature of the interactions established. More negative enthalpy variations reflect the establishment of favorable interactions with the membrane, whereas associations favored by an increase in entropy are mostly due to the hydrophobic effect [31,32,33,34,35]. 

The experimental results were complemented by Molecular Dynamics (MD) simulations. Quantum mechanical calculations were carried out for all different possible isomers of H33342 with overall charges 0, +1, and +2, enabling the determination of the most stable one in each case. These selected isomers were then simulated in the presence of POPC bilayers, to acquire molecular detail of the interactions established [36,37,38]. Though interactions of H33342 with proteins [39,40,41,42] or DNA [43] have been previously characterized using MD simulations, this powerful methodology has, to our best knowledge, not yet been applied to the interactions between H33342 and lipid membranes. In the present work, we use unrestrained MD simulations to characterize several properties of the interaction of H33342 at different protonation states with POPC membranes, namely, transverse localization, preferred orientation of the solute in the bilayers, and the possibility of hydrogen bonding between solute and lipid bilayer components. Umbrella sampling (US) simulations were also performed to obtain the Potential of Mean Force (PMF) across the POPC bilayer, providing insights on the effect of protonation on the rate of permeation through the membrane.

Altogether, this work provides the identification of key properties that allow for H33342 to interact efficiently with cell membranes and to have a fast permeation despite its high global charge. The exploration of these properties in drug design may contribute to the development of new drugs with improved bioavailability.

## 2. Results and Discussion

### 2.1. Partition to POPC LUVs

**Figure 2:**
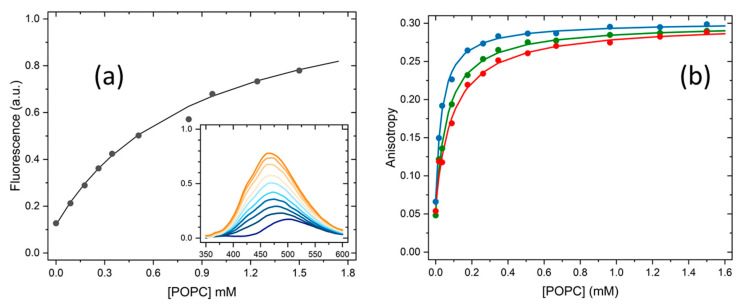
Variation in H33342 fluorescence intensity at 470 nm (**a**) and fluorescence anisotropy at 450–470 nm (●), 470–490 nm (●), and 490–510 nm (●) (**b**) with the concentration of POPC at pH = 6. The values obtained for the partition coefficient were all similar, leading to 1.8(±0.6) × 10^3^. The average value obtained for all samples (N = 6) and conditions is shown in Table 1.

As explained in detail in Section 3.5, the partition of H33342 to POPC LUVs was characterized from the variation in the fluorescence intensity and from the variation in the fluorescence anisotropy. At pH ≥ 6, association with the membrane leads to a large increase in the fluorescence intensity, allowing for directly obtaining the amount of H33342 in both media. However, for pH ≤ 6, the major species in the aqueous media has a large fluorescence quantum yield, and only a small variation is observed upon association with the membrane. Under these conditions, the quantification of the partition coefficient from the variation in the fluorescence intensity has a large uncertainty. On the other hand, interaction with the membrane imposes constraints in H33342 mobility, leading to a significant increase in the fluorescence anisotropy. The variation observed in the fluorescence anisotropy at a given volume of membrane is, however, not a direct measure of the amount of H33342 in each media. Instead, it reflects the relative fluorescence intensity from the probe molecules in both media. To validate the methodology based on the fluorescence anisotropy, both approaches were followed at pH = 6. Typical results obtained at this pH are shown in Figure 2. The fluorescence anisotropy depends very strongly on the POPC concentration, because the anisotropy is dominated by the H33342 associated with the membrane. That is, the ratio between the fluorescence intensity from the probe in the membrane and that from the probe in the aqueous phase ($\alpha_{M}$) is much larger than 1. This parameter is larger at shorter wavelengths, because the spectrum of the probe associated with the membrane is blue-shifted when compared to that for the probe in the aqueous media. Thus, the fluorescence anisotropy increases more abruptly with the lipid concentration for fluorescence detection at lower wavelengths (Figure 2b). When the variation in the fluorescence anisotropy is weighted by $\alpha_{M}$ ($\alpha_{M}=1$3 in the range of 450–470 nm, 8 in the range of 470–490 nm, and 5 in the range of 490–510 nm), the obtained partition coefficient is essentially independent of the detection wavelength. Statistically equivalent values are also obtained from the analysis of the fluorescence intensity (Equation (2)) and the fluorescence anisotropy (Equation (3)).

**Figure A2:**
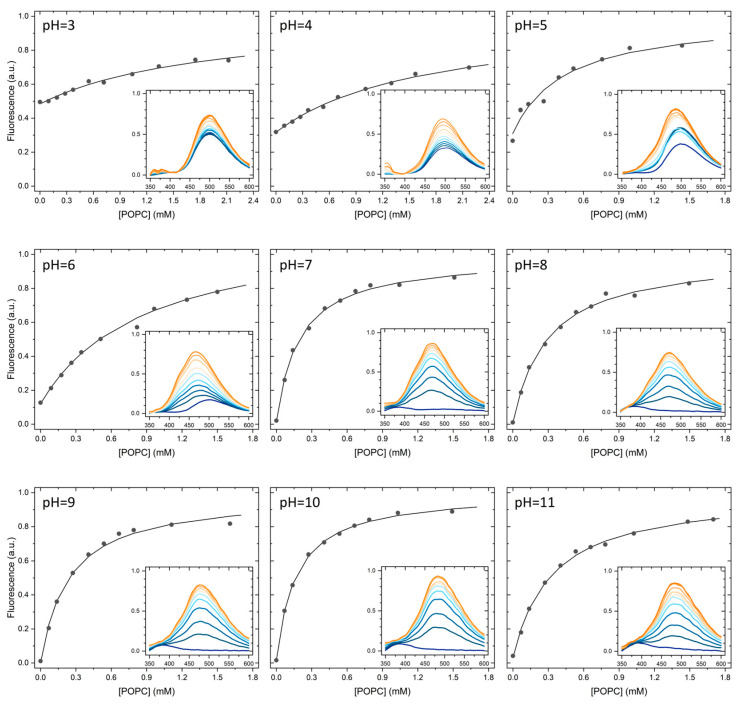
Typical results obtained for the variation in H33342 fluorescence emission intensity with the concentration of POPC in LUVs at different pH values. The lines are the best fit of a simple partition, Equation (2).

**Figure A3:**
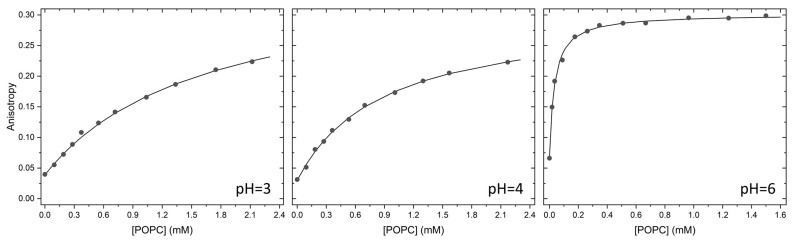
Typical results obtained for the variation in H33342 fluorescence anisotropy (λ_exc_ = 340 nm, λ_em_ = 500–510, 450–510, and 450–470 nm for pH = 3, 4, and 6, respectively) with the concentration of POPC in LUVs at different pH values. The lines are the best fit of a simple partition, Equation (3), with $\alpha_{M}=2$, 2, and 13 for pH = 3, 4, and 6, respectively.

Typical results obtained at all pH values studied are shown in Figure A2 and Figure A3 of Appendix A, and the average values of *K*_P_ obtained are provided in Table 1. The partition coefficient obtained at neutral pH is in excellent agreement with the values reported in the literature for partition to DMPC membranes in the liquid disordered phase [10], as well as to the lipid bilayer of inside-out vesicles of *Lactococcus lactis* [44]. A maximal value of *K*_P_ is observed at pH = 10, in agreement with the higher abundance of the neutral form, predicted in literature studies [12,45,46] and also by the MarvinSketch software [24]. The affinity for the POPC bilayers is essentially unaffected by the pH when the latter varies between 7 and 11, although it is predicted that at pH = 7, the most abundant species is the monocation. A significant decrease is observed for acidic pH values. However, this variation is still relatively modest, with only one order of magnitude decrease between pH = 10 and 3. This result is unexpected, given that the most abundant species is +3 at pH = 3. A relatively high membrane affinity of cationic amines towards phosphatidylcholine membranes has been reported before and was interpreted as ionic interactions with opposite charges in the membrane lipids, also present in zwitterionic membranes [47,48]. However, a significant decrease in membrane affinity is obtained for negatively charged species [49,50,51], pointing towards other important aspects of the interaction of cationic amines with lipid bilayers [47,48].

**Figure 3:**
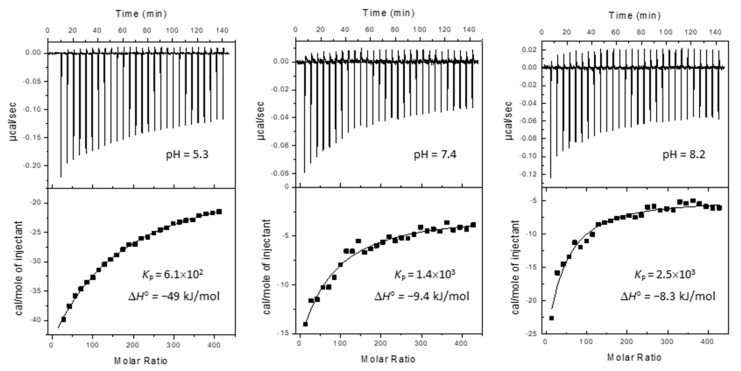
Titration curves and best-fit parameters obtained for the titration of H33342 (5 µM in the ITC cell) with 10 µL additions of POPC LUVs (10 mM in the ITC syringe), at 25 °C in PBS containing 2% (*v*/*v*) DMSO and at the pH values indicated.

To understand the effect of pH on the partition of H33342 to the POPC membranes, the enthalpy of interaction was characterized by ITC. The enthalpy variation obtained directly from the thermogram includes contributions from the interactions established between the solute and the medium where it is dissolved (aqueous medium or membrane), as well as from changes in solute ionization upon partition, the latter also including contributions from changes in the ionization of the buffer [32,52,53]. The phosphate buffer used in the ITC experiments has a very low ionization enthalpy [54], thus having a negligible contribution to the observed enthalpy variation. Therefore, the overall value obtained for the enthalpy variation reflects only changes in H33342, interactions established with the aqueous medium and the lipid membrane, and possible changes in ionization upon partition. Figure 3 shows typical titration curves obtained at different pH values. The partition coefficients obtained are in good agreement with those obtained from changes in H33342 fluorescence properties, although somewhat lower. This may be due to the higher concentration used in the ITC experiments, to incomplete equilibration of H33342 between the two bilayer leaflets during the titration [31], or to a small preference for membranes with a higher curvature [55]. This was not further explored, because the enthalpy variation is the most relevant parameter from those experiments.

It is observed that the interaction of H33342 with the POPC membranes shifts from being stabilized by enthalpy at acidic pH values (Δ*H*^o^ = −54 ± 7 kJ/mol and *T*Δ*S*^o^ = −38 kJ/mol at pH = 5.3) to an equal contribution from enthalpy and entropy at alkaline pH values (Δ*H*^o^ = −9 ± 2 kJ/mol and *T*Δ*S*^o^ = 9 kJ/mol at pH = 8.2). This suggests that the association of the neutral form of H33342 with POPC membranes is mostly stabilized by the hydrophobic effect, and stabilizing electrostatic interactions are established between the cationic forms of H33342 and the membrane, even in the case of membranes containing only zwitterionic lipids such as POPC. To further elucidate the nature of the interactions established, computational studies were performed with H33342 in distinct protonation states. The results obtained are presented in the next sections.

### 2.2. Quantum Chemical Determinations of Isomer Relative Stability

**Figure 4:**
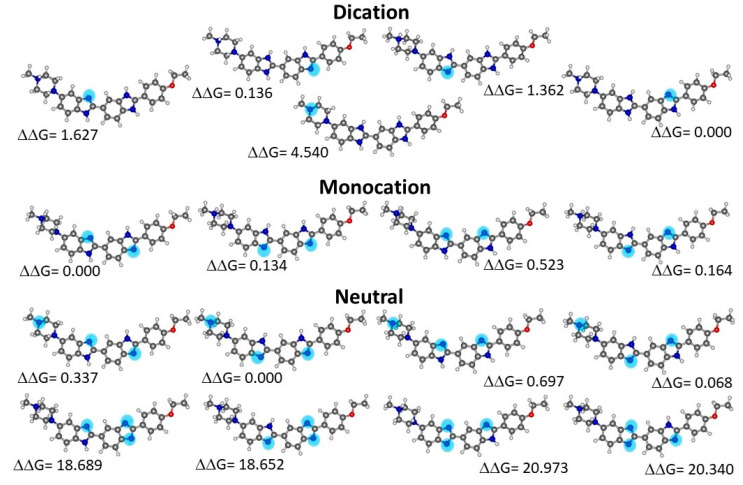
Structures of Hoechst 33342 species in water optimized at PCM-B3LYP/6-31G(d,p) level. Relative free energy values are in kcal mol^−1^. Non-protonated nitrogen atoms are highlighted.

Geometry optimizations were conducted for the monocationic form of H33442 (H(+1)), its neutral form (H(0)), obtained by the removal of one proton, and for the doubly charged molecular cation (H(+2)), obtained by further protonation relative to the monocation. For each of the different charge values, there are several protonation/deprotonation possibilities corresponding to different isomers. From a total number of 17 molecular species, the most stable structures of the neutral, mono-charged, and double-charged cations, respectively, were selected by calculating their relative Gibbs energy in solution and were used to conduct the MD simulation. Figure 4 presents the optimized isomers thus obtained, together with relative free energies in aqueous solution.

### 2.3. Unrestrained MD Simulations

**Figure 5:**
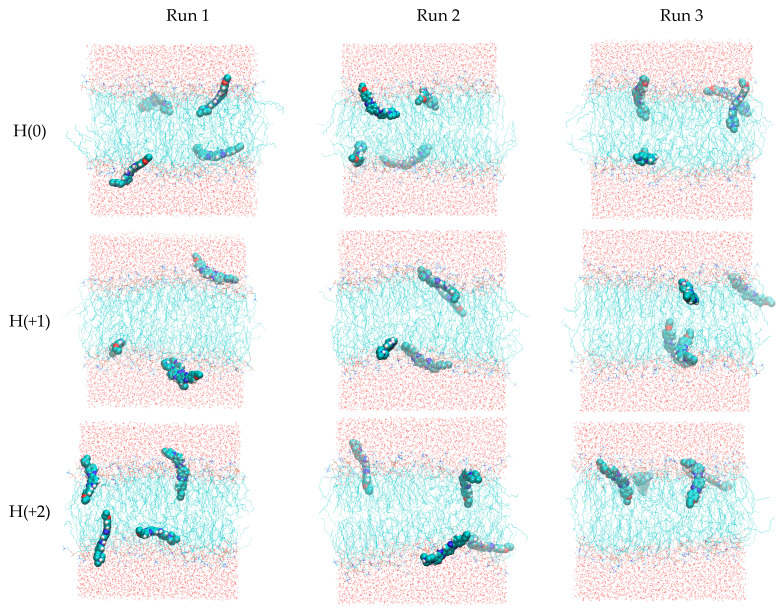
Final snapshots of production runs. Each row concerns a different ionization state (H(0), H(+1), and H(+2), respectively). Different columns refer to runs 1, 2 (with probes starting from water), and 3 (with probes starting in the bilayer midplane) in each ionization state, respectively.

As described in the methods section, the single most stable structures obtained from the quantum chemical calculations for H(0), H(+1), and H(+2) were parameterized for MD simulations and simulated in triplicate for 200 ns, in the presence of a POPC bilayer. Figure 5 depicts final snapshots in each simulation (run 1 and 2 refer to the system with probe molecules initially placed in the water phase, and in run 3, the probe molecules were initially placed in the bilayer midplane). Even though they only depict a single configuration state, some general features can already be observed. All molecules, irrespective of the ionization state, interact with the lipid bilayer. H(+2) molecules often adopt an orientation perpendicular to the membrane plane, where they insert the region of the molecule bearing no protonated sites (ring 3, ethoxy substituent) into the bilayer, while keeping the protonated region (ring 1, piperazine) in the upper headgroup region or even protruding into the water. This tendency is progressively lost for lower ionization states. Figure 5 reveals that two molecules in run 1 of H(+1) aggregate (average distance between centers of mass is 0.90 nm in the last 150 ns of simulation, with minimal distance of 0.36 nm; the dimer is clearly visible in the lower leaflet). Aggregation markedly affects probe interactions with the bilayer, and because we believe that this is a result of the relatively high probe:lipid ratio (1:32) necessary for simulating an adequate number of solute molecules, we excluded these two molecules from subsequent analyses. Therefore, whereas in the H(0) and H(+2) analyses, twelve molecules were used for averaging, only ten were considered for H(+1).

The time evolution of the center of mass vertical position (*z*) for the four individual molecules in each run (Figure S1 in the Supplementary Material file) shows that *z* converges rapidly in the simulations that start with the molecules in the bilayer midplane (*z* = 0, replicate 3), mostly to values in the 1 nm < |*z*| < 1.5 nm range. However, in replicates 1 and 2, where H33342 molecules were initially placed in the water/headgroup interface, insertion of some molecules is still occurring after 50 ns. Some molecules in these systems appear to not have actually inserted by the end of the simulations, residing at |*z*| ≈ 2 nm. Despite these different behaviors, all molecules (except the aggregated pair in run 1 of H(+1), as previously explained) were included for subsequent analyses, after discarding the first 100 ns of each simulation.

**Figure 6:**
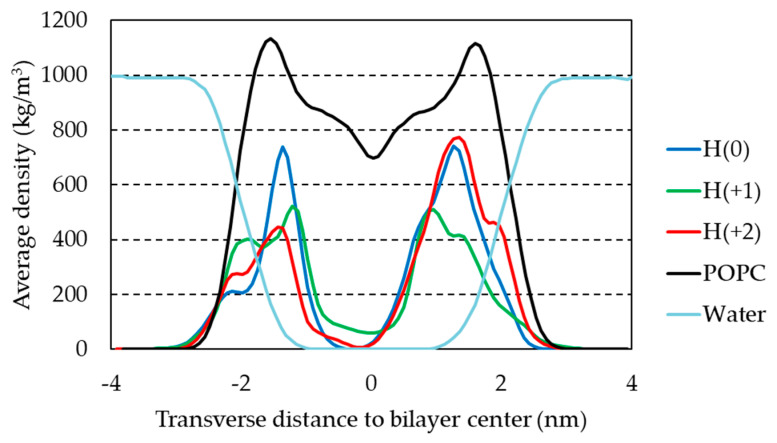
Mass density profiles of H33342 (multiplied by a factor of 20 for better visualization) along the direction normal to the bilayer plane, for the three studied forms. The shown curves are the average of the behavior of all molecules (except the aggregated molecules in run 1 of H(+1)) across the last 100 ns of the different simulations. For reference, also shown are the profiles obtained for POPC and water in probe-free simulations.

The mass density of H33342 along the *z* direction is shown in Figure 6 for the three ionization states. Overall, no pronounced differences are observed, with the preferred location being around 1.5 nm from the bilayer center for all forms. This was already suggested by the similar position of the center of mass (Figure S1). Curiously, H(+1) shows a more internal peak than both H(0) and H(+2), but it has a wider density profile. 

**Figure 7:**
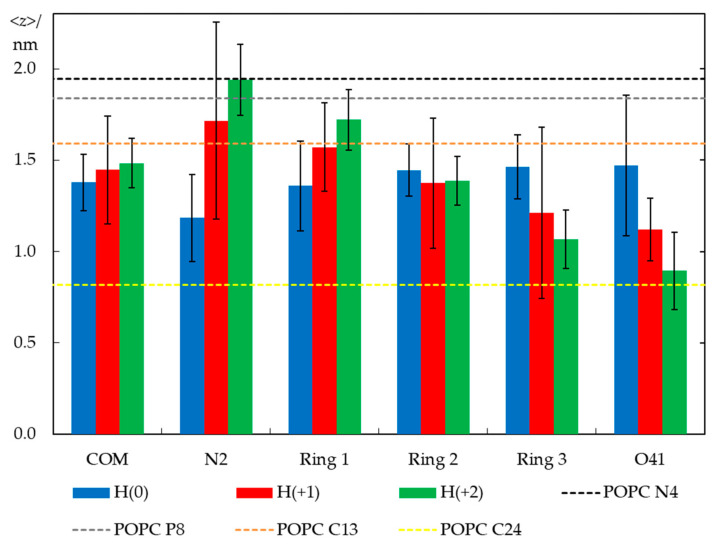
Average transverse distances to the bilayer center for the center of mass (COM) of different atoms/groups (defined in Figure 1) of H33342 (in columns) and, as a reference, for different lipid atoms in probe-free bilayers (in dotted lines). Averaging and error estimation involved the 12 H33342 molecules (10 for H(+1)) simulated in the three replicates of each ionization state.

**Figure 8:**
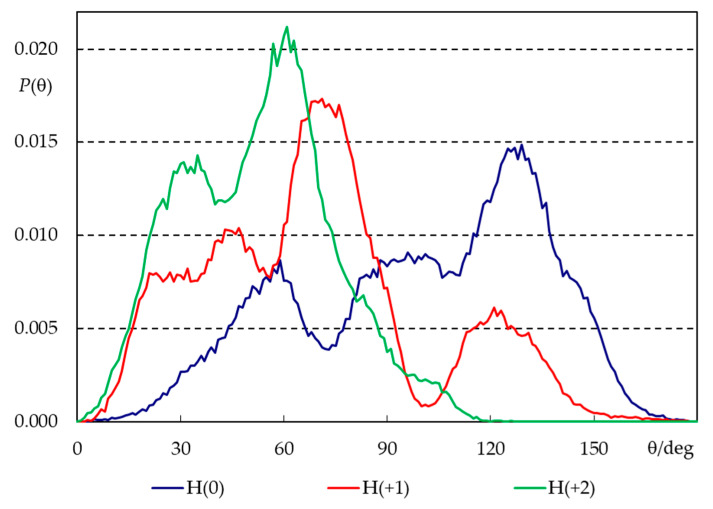
Angular distributions for the tilt of H33342 long axis, relative to the bilayer normal, for the three simulated ionization states (see Figure 1a for axis definition).

Despite this similarity, the molecules clearly change their average orientation in the bilayer in response to varying their degree of protonation. We looked at orientation differences in two ways: by calculating average positions of different structural groups (Figure 7) and angular distributions for the H33342 long axis (defined in Figure 1a) tilt relative to the bilayer normal (Figure 8).

Figure 7 shows average transverse positions of groups and atoms of H33342, in comparison with those determined for selected atoms of POPC in a probe-free system (as shown in Figure S2, the presence of H33342 leads to very slight changes in POPC atom locations, <1.5% for those included in Figure 7). Globally, it illustrates that the probe has similar vertical localization in the membrane for all ionization states, corresponding to the upper acyl chain/carbonyl/glycerol region of the bilayer. The center of mass position appears to show a very slight dependence on the ionization state, and slightly more external locations are observed for progressively increased overall charge. However, the difference is ultimately very small and well within the estimated uncertainty, in accordance with the density profile analysis above. Still, there is a larger difference in positions of particular H33342 atoms/groups, which, though not entirely significant at the 95% confidence level considered in the figure error bars, appears to be sufficiently consistent overall to allow the following considerations. The uncharged species prefers to adopt an almost horizontal orientation, in the plane of the bilayer. The average positions of the different atoms of H(0) lie in a narrow bilayer region (between ~1.2 and 1.5 nm) for this ionization state, with the piperazine ring (N2) slightly more internal than other parts of the molecule. This contrasts with the behavior observed for H(+2). For this species, the most internally located part is the ethoxy group, with the O41 atom located on average at *z* = (0.89 ± 0.21) nm. Conversely, the opposite end of the molecule (the piperazine ring) has a very external transverse location in the headgroup region (*z* = (1.94 ± 0.19) nm), implying that in this ionization state, H33342 spans ~1 nm in the direction normal to the bilayer plane. An intermediate scenario is observed for H(+1), with specific atoms/groups located on average between *z* = (1.12 ± 0.17) nm (for O41) and (1.71 ± 0.54) nm (for N2).

The long axis tilt distributions (Figure 8) confirm these patterns. All distributions are very wide, illustrating the flexibility in the orientation of H33342 in the bilayer as well as distinct behaviors of different molecules (some of which actually do not insert properly in the course of the simulation, as pointed out above). Still, the widest distribution is clearly that corresponding to H(0), which displays significant probability density both for θ < 90° (piperazine pointing towards water) and θ > 90° (piperazine pointing towards center of bilayer), though the latter dominates slightly, and the average tilt angle for this state is <θ> = 100°. The opposite behavior is verified for H(+2). This form favors the orientation of piperazine towards water and the insertion of the opposing end, with <θ> = 52°. Again, H(+1) displays intermediate behavior (<θ> = 74°).

**Figure 9:**
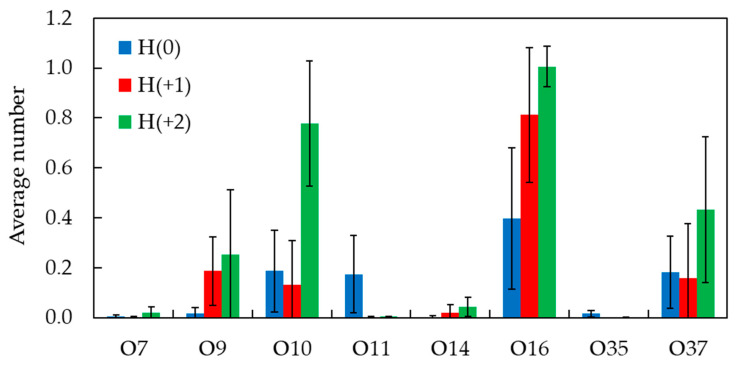
Fraction of time of hydrogen bonds between H33342 N-H donor groups and POPC oxygen atoms.

To obtain further details on the interactions established between H33342 and the lipid molecules and how they may depend on the probe ionization state, we studied the fractional frequency of hydrogen bonding between NH donor groups of H34442 and oxygen acceptor atoms of POPC (Figure 9). Globally, it is observed that H bonding to glycerol O atoms (O14 and O35 for *sn*-2 and *sn*-1 lipid acyl chains, respectively) is low in all cases (<2% of total for the three forms), and only bonding to POPC O atoms from the phosphate (O7, O9, O10, and O11, mainly the three latter atoms) and carbonyl (O16 and O37 for *sn*-2 and *sn*-1 lipid acyl chains, respectively) groups needs to be considered.

It must be noted that the higher the probe charge, the larger the number of NH donors available for H bonding. H(0) has two NH groups, one in each of the benzimidazole rings. H(+1) has one more NH group, in the piperazine ring, and H(+2) has that group and an additional one, in the benzimidazole ring closest to the piperazine. The fractional frequency of H bonding summed across all POPC acceptor atoms reflects this obvious fact and increases in the order 0.98 (H(0)) < 1.31 (H(+1)) < 2.53 (H(+2)). When these values are divided by the actual number of NH donor groups, similar values are obtained for H(0) and H(+1) (0.49 and 0.44, respectively), whereas the one for H(+2) is slightly higher (0.63). Overall, these results indicate that H33342 NH groups are roughly involved in H bonding to lipid O acceptors 40–60% of the time. The fractions of bonding to the phosphate (39%, 25%, and 41% for H(0), H(+1), and H(+2), respectively) or carbonyl POPC O atoms (59%, 74%, and 57% for H(0), H(+1), and H(+2), respectively) are similar for the three forms. The fact that maximal bonding to the phosphate occurs for H(+2) (41%) reflects perhaps the more external position of piperazine and benzimidazole ring 1, all with NH donors. However, in general, the transverse location of the NH donor groups is around *z* ~1.5 nm (Figure 7), enabling efficient H bonding to carbonyl POPC atoms, which are the dominant interactions.

The higher number of hydrogen bonds from H33342 NH to POPC acceptor atoms, as H33342 protonation increases, may be correlated with the increase observed in the interaction enthalpy as the pH is decreased (Figure 3). Those favorable interactions can also explain the relatively small variation observed in the affinity for the membrane, despite the large decrease in the hydrophobicity of the protonated charged forms of H33342.

### 2.4. Free Energy Profiles from Umbrella Sampling MD

**Figure 10:**
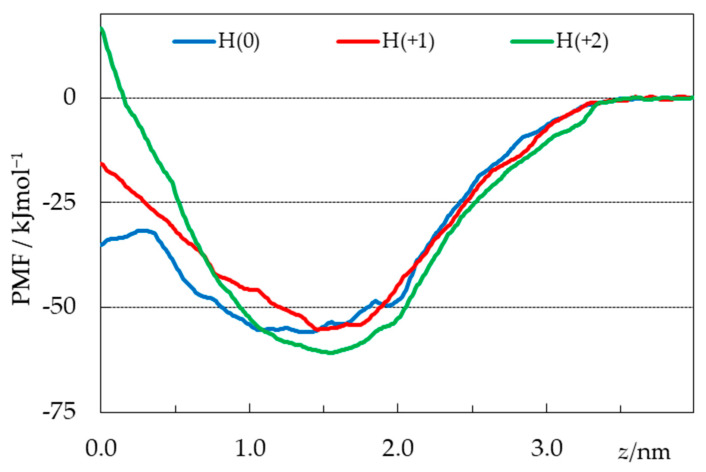
PMF profiles of the simulated H33442 forms (obtained discarding the first 40 ns from analysis), as a function of the distance *z* between the centers of mass of the probe and the bilayer.

From umbrella sampling simulations, free energy profiles were calculated for the three considered H33442 forms, as described in detail in the Methods section. The potential of mean force (PMF) curves are shown in Figure 10, whereas Figures S3–S5 depict uncertainty estimates and convergence analysis (PMF profiles obtained using different regions of the 0 < *t* < 120 ns simulation time range, following the procedure of Reference [57]) for the different curves.

As expected for amphiphilic solutes [57,58,59,60,61], the free energy is reduced when they interact with the lipid bilayer, coming from the aqueous medium. Energy minima are observed for the three ionization states at depths close to those obtained from the unrestrained simulations and shown in Figure 7, again similar for all forms (slightly more interior for the neutral form). The minimal ordinate values are also similar for the three states. Whereas the value calculated for the H(+2) form is lower than those obtained for the other ones by ~5–6 kJmol^−1^, this difference is smaller than the uncertainty of approximately ±10 kJmol^−1^ indicated from bootstrapping analysis (Figures S3E–S5E). In the absence of clearcut distinction in the regions around the equilibrium locations, the most striking difference in the three free energy profiles occurs for the regions nearest the center of the bilayer (*z* ≈ 0). Neutral H33442 displays a 24 kJmol^−1^ energy barrier for crossing from the free energy minimum to a maximum located at *z* = 0.26 nm, before decreasing by 3.5 kJmol^−1^ between the latter location and *z* = 0. In recent studies by our group, free energy plateaus or slight decreases near the bilayer center have been obtained for neutral solutes such as cholesterol [58], neutral rhodamine dyes [59], or undissociated fatty acids [60]. This feature is not observed in the profiles of the charged H(+1) and H(+2), for which maxima are observed at *z* = 0, with free energy barriers (of 40 and 78 kJmol^−1^, respectively) that increase with the charge of the solute.

Following the procedure used in recent studies [59,62,63,64,65,66], these calculated free energy profiles may be used to obtain computational estimates of the partition coefficient:

(1) $$K_{P}\;\int_{0}^{a}exp(-\Delta G\left(z\right)/RT)dz$$

Because of different underlying reference states in the experimental and calculated *K*_P_, as well as arbitrariness in the location of the bulk water/lipid interphase in the simulations, here considered to be *a* = 4.0 nm, no attempt is made to calculate absolute *K*_P_ values. Still, we can compare relative values of computational estimates of *K*_P_ for the three simulated forms. From the PMF profiles, the lowest *K*_P_ value was obtained for the H(+1) state, below the corresponding ones for H(0) and H(+2) by 0.3 and 1.0 log units, respectively. This order does not fully coincide with the one resulting from the experimental determinations (Table 1), which point to a steady ~two-fold (or 0.4 log units) increase in *K*_P_ as pH is increased from 5 to 9, with the dominant protonation state varying between +2 and 0 [12,45,46]. Still, both experimental and computational values agree in the fact that *K*_P_ shows low sensitivity across a wide range of pH values.

## 3. Materials and Methods

### 3.1. Materials

The lipid 1-palmitoyl-2-oleoyl-*sn*-glycero-3-phosphocholine (POPC) was acquired from Avanti Polar Lipids, Inc. (Alabaster, AL, USA). Hoechst 33342 (H33342) was acquired from Sigma-Aldrich Química S.A. (Sintra, Portugal). The water used to prepare the solutions was first distilled, and then deionized by ion exchange cartridges and activated carbon filters (ARIOSO UP, from Human, Seoul, Republic of Korea), with a final resistance of ≥18 MΩ. Reagents and solvents used were analytical-grade or of higher purity.

### 3.2. Preparation of Large Unilamellar Vesicles (LUVs)

The LUVs were prepared by extrusion as previously described [67]. For the partition experiments followed by H33342 fluorescence properties, the aqueous buffer used to hydrate the lipid film contained 3.3 mM Trizma, 3.3 mM citric acid, 3.3 mM ammonium phosphate, 150 mM NaCl, and 0.02% *m*/*v* sodium azide, with the pH previously adjusted to the required value by addition of HCl or NaOH (pH between 3 and 11). Large Unilamelar Vesicles (LUVs) were prepared from the MLVs obtained after hydration of the lipid film by freeze–thaw cycles and extrusion through two stacked polycarbonate filters (with a pore diameter of 100 nm (for ITC experiments) or 50 nm (for fluorescence experiments). The smaller size used in the fluorescence-based experiments was required to decrease the contribution from light scatter. The liposomes were allowed to stabilize overnight before use, to allow for the dissipation of the stress imposed by extrusion, and their effective size was analyzed by dynamic light scattering (Zetasizer Nano ZS, Malvern). The average diameter was 77 ± 2 nm or 112 ± 7 nm when filters with a pore diameter of 50 or 100 nm were used for extrusion, respectively. The final lipid concentration was quantified using the Bartlett method. The liposome samples were stored at 4 to 8 °C and used within 2 weeks.

### 3.3. Partition of Hoechst 33342 to POPC LUVs

A stock of H33342 was first prepared in methanol and quantified by UV-vis absorption (Spectronic Unicam UV500) assuming a molar absorptivity of 4.7 × 10^4^ M^−1^ cm^−1^ at 343 nm [68]. The required volume of this solution was then evaporated to prepare a stock of H33342 in DMSO at a concentration of 50 µM. An aliquot from this stock was squirted under vortex to a buffer solution (previously heated to 37 °C), to obtain a final solution with 2% DMSO and Hoechst 33342 at 1 µM. The fluorescence intensity spectra and/or the fluorescence anisotropy were obtained (Carry Eclipse, Varian, Palo Alto, CA, USA) for excitation at 340 nm. Aliquots of the LUV suspension were then added, and the fluorescence signal was obtained, with 10 min equilibration between each LUV addition.

At pH ≥ 6, partition of H33342 to the lipid bilayer leads to a strong increase in the fluorescence intensity and a red shift in the spectra. The partition coefficient (*K*_P_) was calculated from the dependence of the fluorescence intensity at 470 nm with the lipid concentration, Equation (2):

(2) $$I=\frac{I_{W}+I_{M}K_{P}{\bar{V}}_{\mathrm{POPC}}\left[\mathrm{POPC}\right]}{1+K_{P}{\bar{V}}_{\mathrm{POPC}}\left[\mathrm{POPC}\right]}$$

where $I_{W}$ and $I_{M}$ are the fluorescence intensity obtained when all H33342 is in the aqueous medium or associated with the membrane, respectively. A molar volume (${\bar{V}}_{\mathrm{POPC}}$) of 0.8 dm^3^/mol was considered, and it was assumed that all lipids are accessible (corresponding to full translocation of Hoechst during the 10 min equilibration).

**Figure 11:**
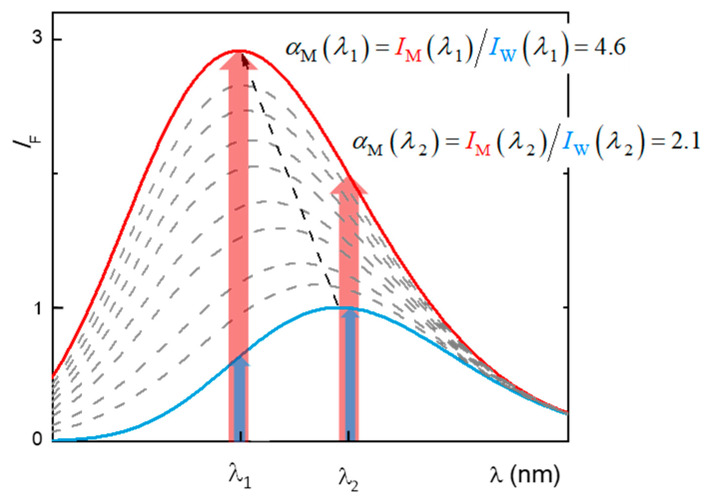
Graphical exemplification of the method used to calculate the parameter $\alpha_{M}$ for use in Equation (3). The continuous lines represent the fluorescence emission spectra of the probe in the aqueous medium (blue) and in the membrane (red), and the dashed grey lines represent the spectra at different lipid concentrations. The highlighted wavelengths ($\lambda_{1}$ and $\lambda_{2}$) correspond to hypothetical wavelengths selected for the use of fluorescence anisotropy to calculate the partition coefficient and are thus where the parameter $\alpha_{M}$ must be calculated.

At lower pH values, the fluorescence intensity from H33342 in the aqueous medium increases significantly with a maximum emission at 500 nm, being similar in both media (aqueous and membrane). Due to the small variation in the fluorescence intensity, the estimates of *K*_P_ obtained from Equation (2) have a large uncertainty. In these conditions, association with the membrane is followed more accurately through the variation in the fluorescence anisotropy, Equation (3):

(3) $$r=\frac{r_{W}+\alpha_{M}\;r_{M}K_{P}{\bar{V}}_{\mathrm{POPC}}\left[\mathrm{POPC}\right]}{1+\alpha_{M}\;K_{P}{\bar{V}}_{\mathrm{POPC}}\left[\mathrm{POPC}\right]}$$

where $r_{W}$ and $r_{M}$ correspond to the fluorescence anisotropy when H33342 is all in the aqueous medium or in the membrane, and *r* is that observed at a given lipid concentration. The parameter $\alpha_{M}$ is the ratio between the fluorescence emission intensity when H33342 is in the membrane and in the aqueous medium, as exemplified in Figure 11. This parameter was estimated from the trend observed in the fluorescence intensity.

The association of H33342 with the POPC membranes was also characterized by Isothermal Titration Calorimetry (ITC) to obtain the enthalpy of the interaction. Titrations were performed on a VP-ITC from MicroCal (Northampton, MA, USA) at 25 °C, with injection speed 0.5 µL/s, stirring speed 459 rpm, and reference power 10 µcal/s. The LUVs (at a lipid concentration of 10 mM) were placed in the syringe and added to H33342 (at a concentration of 5 µM) in 10 µL aliquots. The solvent was saline phosphate buffer (10 mM phosphate with 150 mM NaCl, previously equilibrated at different pH values from 5.3 to 8.2) containing 2% (*v*/*v*) DMSO. All solutions were previously degassed for 10 min. The thermogram was integrated using the data analysis software Origin 7.0 as modified by MicroCal for ITC experiments. The heat evolved was analyzed assuming simple partition and considering that all lipids are available for interaction with H33342. The fitting parameters, $K_{P}$ and $\Delta H^{o}$, were obtained using the Solver Add-In from Excel^®^ with the spreadsheet provided in Reference [69].

### 3.4. Quantum Chemical Calculations

Geometries of H33442 in different protonated forms were obtained by density functional theory (DFT) from optimizations using the hybrid exchange–correlation functional B3LYP [70,71] with the 6-31G(d,p) basis set. Solvent effects were considered by means of the implicit polarized continuum (PCM) [72,73] model. Frequency analysis confirmed that each optimized geometry was an energy minimum by the non-existence of imaginary frequencies. Free energies in solution were calculated at room temperature (*T* = 298 K), with the thermal corrections calculated using the quasiharmonic oscillator approximation, where vibrational frequencies smaller than 100 cm^−1^ are raised to 100 cm^−1^ due to the failure of the harmonic oscillator model for low-frequency vibrational modes [74].

Partial charges in selected H33442 in different protonated forms were calculated from the optimized geometries, followed by a least-squares fit to the electrostatic potential obtained at the same theory level, according to the Merz-Singh-Kollman schemes [75,76]. All quantum calculations were performed with the Gaussian 16 package [77].

### 3.5. MD Simulation Details

MD simulation and trajectory analysis was carried out using GROMACS 5 [78,79,80]. The united-atom GROMOS 54a7 force field was used [81], with lipid parameters from Poger and Mark [82,83]. For H33342, the most stable structures obtained from the quantum chemical calculations for H(0), H(+1), and H(+2) were modeled using the Automated Topology Builder (ATB) [84,85,86]. We replaced the atomic charges generated by ATB with the values obtained as described in the previous subsection. The stability of the probe force field parameters was verified in simulations of each of three ionization states in water (not shown). A fully hydrated POPC bilayer, composed of 128 lipids and 5412 SPC waters [87], was built with standard GROMACS tools. For each ionization state, three different systems were generated and simulated, all of them with all four H33342 molecules. In two of these replicates (1 and 2 in Figure 5 and Figure S1), the probes were initially placed just outside the bilayer, 2 near each leaflet. In the other one (referred to as 3 in Figure 5 and Figure S1), H33442 molecules were placed in the POPC bilayer midplane. In the simulations with charged H33342, a corresponding number of chloride ions was added to the aqueous medium, to ensure electroneutrality.

All systems were simulated under *NpT* conditions at 298 K and 1 bar. Equilibration/production run protocols and other simulation options were conducted as described elsewhere [88]. Each of the nine production runs was carried out for 200 ns. For visualization of structures and trajectories, VMD software (version 1.9.3.) was used [89].

The free energy of the system (Δ*G*), as a function of the reaction coordinate (defined as the distance between the center of mass of the H33442 molecule and the local center of mass of the POPC bilayer, i.e., calculated using only the POPC molecules whose locations in the bilayer plane were contained in a 1.1 nm radius cylinder centered on the solute), is derived from the PMF, obtained from a series of umbrella sampling (US) simulations [90]. For this purpose, a solute molecule, initially in the aqueous phase, is first pulled to the center of the bilayer (*z* = 0), with a rate of 0.0005 nm/ps and a force constant of 500 kJmol^−1^ nm^−2^. Subsequently, a second pulling run was carried out, gently pulling in the opposite direction, using the same speed and force constant of the previous step, starting from *z* = 0 and ending at *z* = 4.0 nm. From this simulation, 41 configurations were extracted in which the molecule was approximately in each of the transverse positions between *z* = 0 and *z* = 4.0 nm, spaced 0.1 nm apart. This procedure was chosen to obtain the initial configurations for the umbrella sampling runs, avoiding the slow convergence of the free energy profiles observed in similar systems, when the final pulling is performed from the water medium to the membrane interior [57]. For the three H33442 forms, each of these 41 systems was simulated for 120 ns, using the same conditions as in the unrestrained runs but imposing a harmonic restraint potential, centered in the reference position, with a force constant of 3000 kJmol^−1^ nm^−2^. The resulting simulations were checked for convergence (Figures S3–S5) and analyzed using the Weighted Histogram Analysis Method [91,92] to produce the PMF profiles, using the last 80 ns of each run for sampling.

## 4. Conclusions

**Figure 12:**
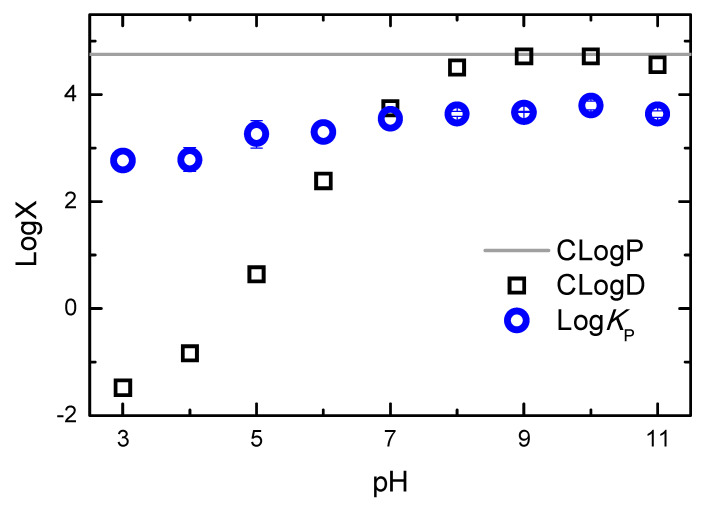
Dependence of the calculated partition (CLogP) and distribution (CLogD) of H33342 between the aqueous medium and octanol, and of the partition coefficient measured experimentally between the aqueous medium and POPC bilayers (Log*K*_P_), as a function of the pH of the aqueous medium. The calculated parameters were obtained with the MarvinSketch software [24].

The results obtained for the association of H33342 with POPC membranes at distinct solute ionization states clearly show that lipophilicity cannot be directly estimated from solute hydrophobicity. The lipids that form biological membranes have a large variety of groups that can interact with the solutes, and biomembranes are very heterogeneous media that may accommodate solutes with distinct physico-chemical properties. This behavior highlights the limitations of the partition between water and water-immiscible non-polar solvents (such as octanol) when estimating the affinity for biomembranes. The limitations of such approaches become strikingly evident when the calculated P and D (octanol/water partition of the neutral form and the distribution of the ionization forms at a given pH value, respectively) are represented together with the observed *K*_P_ as a function of pH for the case of H33342, Figure 12.

The calculated distribution between the aqueous media and octanol is close to the calculated partition coefficient between the two homogeneous solvents in the pH range of 9–10 due to the higher abundance of the H33342 neutral form, and it decreases for lower and higher pH values where the charged forms become more abundant. A decrease of over five orders of magnitude is observed for LogD when the pH is changed from 9 to 3 (where forms +3 and +2 are the most abundant), in agreement with the pH partition hypothesis that assumes that charged species have a negligible partition to non-polar media [93]. In contrast, the partition coefficient between the aqueous media and POPC bilayers decreases by only one order of magnitude in this pH region, as previously observed for other cationic molecules with ionized amine groups [47,48].

The results obtained with the MD simulations allow for the interpretation of the difference between the partition to the homogeneous octanol solvent or to the heterogeneous and complex lipid bilayer. On the one hand, the distinct environments of the lipid bilayer are able to efficiently solvate solutes with distinct physico-chemical properties. On the other hand, glycerophospholipids (the most abundant in biomembranes) contain several charged groups and oxygens that may participate in electrostatic interactions. When amine groups become protonated, the decrease in hydrophobicity provided by the increase in charge is compensated for by an increase in the number of hydrogen bonds that may be established with the acceptor groups in the membrane lipids, leading to small variations in the overall affinity for the membrane. 

In this work, a simplified model membrane composed of POPC was used. This is one of the most abundant lipids in biomembranes [25,26,27,28,29,30] and was selected to represent zwitterionic lipids in the fluid phase. Biomembranes contain a large variety of other lipids, including significant amounts of negatively charged lipids. The presence of these lipids would further reduce the already small decrease observed in the partition coefficient of the cationic species when compared with the neutral form. It should be noted, however, that at the high ionic strength of biological fluids, only a small contribution is expected from electrostatics to the overall interaction affinity (e.g., [31]).

In conclusion, to estimate the local concentration of H33342 in lipid membranes, the overall hydrophobicity estimated from the water/octanol partition cannot be used. The partition coefficients to the actual membranes or to representative model membranes must be used instead. This procedure must be followed whenever the association of solutes with membrane proteins is considered [94], as is the case for the interaction with the efflux transporter P-glycoprotein, of which Hoechst dyes are well-known substrates [13,14]. Failure to do so will lead to apparent affinities for the membrane proteins that do not reflect the interactions established between the ligand and the protein [95].

## Figures and Tables

**Table 1:** Partition coefficient of H33342 towards POPC LUVs at different pH values. The average and standard deviation were calculated for the log *K*_P,_ because this is the variable with a normal distribution [56]; the average *K*_P_ was then calculated from the average of log *K*_P_. The number of replicates (N) for each situation is indicated below the pH value.

	pH	3	4	5	6	7	8	9	10	11
	**N**	4	5	3	6	6	4	2	4	4
**log *K*_P_**	**µ**	2.8	2.8	3.3	3.3	3.6	3.6	3.7	3.8	3.6
	**σ**	0.16	0.22	0.26	0.17	0.19	0.05	0.01	0.08	0.06
***K*_P_**	**µ**	5.8 × 10^2^	6.1 × 10^2^	2.6 × 10^3^	2.0 × 10^3^	3.8 × 10^3^	4.4 × 10^3^	4.7 × 10^3^	6.3 × 10^3^	4.3 × 10^3^

## Data Availability

Source experimental and simulation data will be provided by the corresponding authors upon request.

## Supplementary Materials

The following supporting information can be downloaded at: https://www.mdpi.com/article/10.3390/molecules28155640/s1, Figure S1: Time variations in the center of mass positions of all individual molecules in each simulation; Figure S2: Average transverse distances to the bilayer center for selected atoms of POPC; Figure S3: PMF error and convergence analysis for H(0); Figure S4: PMF error and convergence analysis for H(+1); Figure S5: PMF error and convergence analysis for H(+2).

## Sample Availability

Samples of the compounds are not available from the authors.

## Appendix A

The relative abundance of the different H33342 ionization states as a function of pH is shown in Figure A1, as calculated from the ionization equilibrium constants calculated using the MarvinSketch software [24].

Typical results obtained for the partition of H33342 to POPC liposomes at all studied pH values, based on the variation in H33342 fluorescence intensity (Figure A2) or on the variation in its fluorescence anisotropy (Figure A3).
