# Supplementary material for: Interaction of Hoechst 33342 with POPC Membranes at Different pH Values
Source: Molecules. 2023 Jul 25;28(15):5640. doi: 10.3390/molecules28155640 (PMC10420284; doi:10.3390/molecules28155640)
Supplement: Supplementary file 1 [file molecules-28-05640-s001.zip › molecules-2509446-supplementary.pdf]

Interaction of Hoechst 33342 with POPC membranes at different pH values

Margarida M. Cordeiro [1,2,†], Hugo A. L. Filipe [1,3,†], Patrícia dos Santos [1,2], Jaime Samelo [1,2], João P. Prates Ramalho [4], Luís M. S. Loura [1,5,6,*] and Maria J. Moreno [1,2,5*]

[1] Coimbra Chemistry Center, Institute of Molecular Sciences (CQC-IMS), University of Coimbra, 3004-535 Coimbra, Portugal; mmc.margarida0@gmail.com (M.M.C); p.santos0495@gmail.com (P.S.); jsamelo@uc.pt (J.S.)
[2] Department of Chemistry, Faculty of Sciences and Technology, University of Coimbra, 3004-535 Coimbra, Portugal
[3] Polytechnic of Guarda, CPIRN-IPG—Center of Potential and Innovation of Natural Resources, 6300-559 Guarda, Portugal; hlfilipe@ipg.pt
[4] LAQV, REQUIMTE, Hercules Laboratory, Department of Chemistry, School of Science and Technology, University of Évora, 7000-671 Évora, Portugal; jpcar@uevora.pt
[5] CNC—Center for Neuroscience and Cell Biology, University of Coimbra, 3004-535 Coimbra, Portugal
[6] Faculty of Pharmacy, University of Coimbra, 3000-548 Coimbra, Portugal
* Correspondence: lloura@ff.uc.pt (L.M.S.L.); mmoreno@ci.uc.pt (M.J.M.)
† These authors contributed equally to this work.

Supplemental Figures

Contents

	Page
Figure S1: Time variations of the center of mass positions of all individual molecules in each simulation	S2
Figure S2: Average transverse distances to the bilayer center for selected atoms of POPC	S3
Figure S3: PMF error and convergence analysis for H(0)	S4
Figure S4: PMF error and convergence analysis for H(+1)	S5
Figure S5: PMF error and convergence analysis for H(+2)	S6

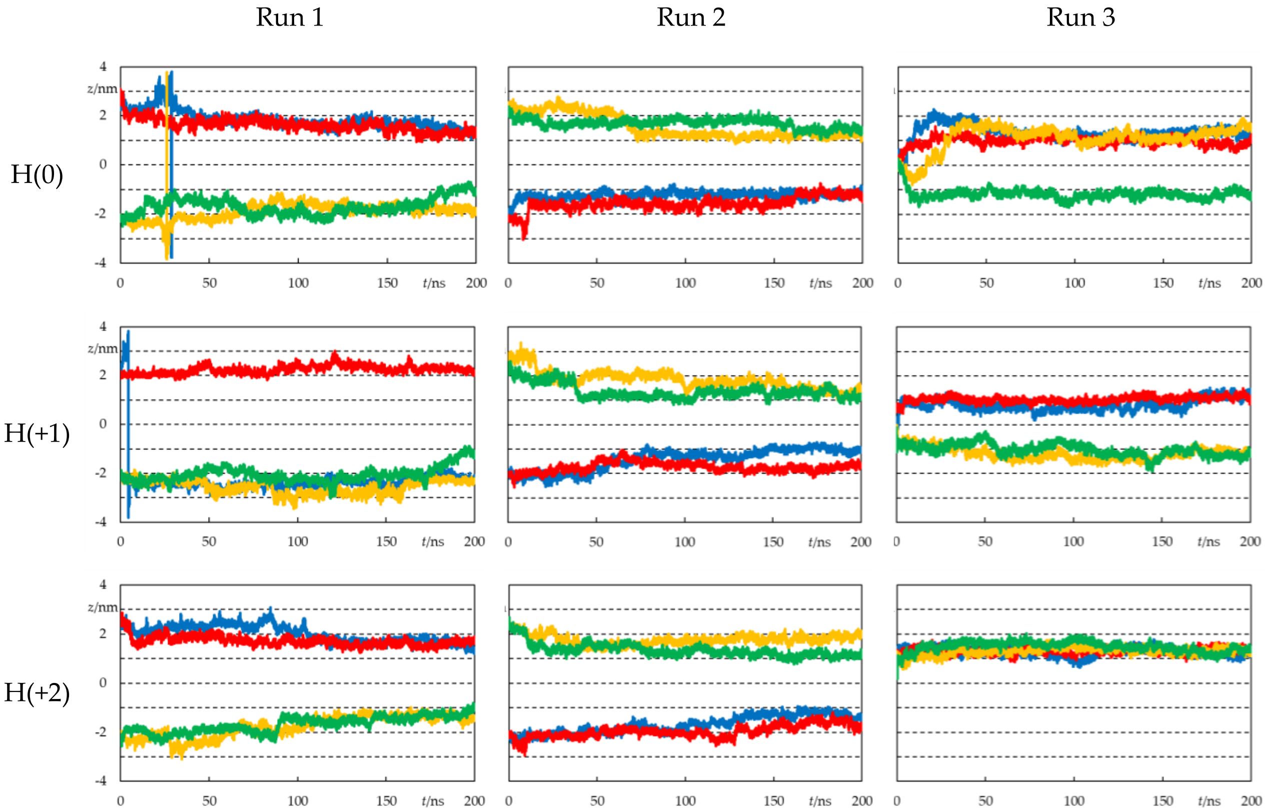

Figure S1. Time variations of the center of mass positions along the bilayer normal, respective to the that of the bilayer center of mass (z = 0), of all four individual molecules in each simulation. Discontinuities in runs 1 of H(0) and H(+1) indicate molecules that diffused through the water medium, crossed the vertical limit of the box and reappeared on the opposite side.

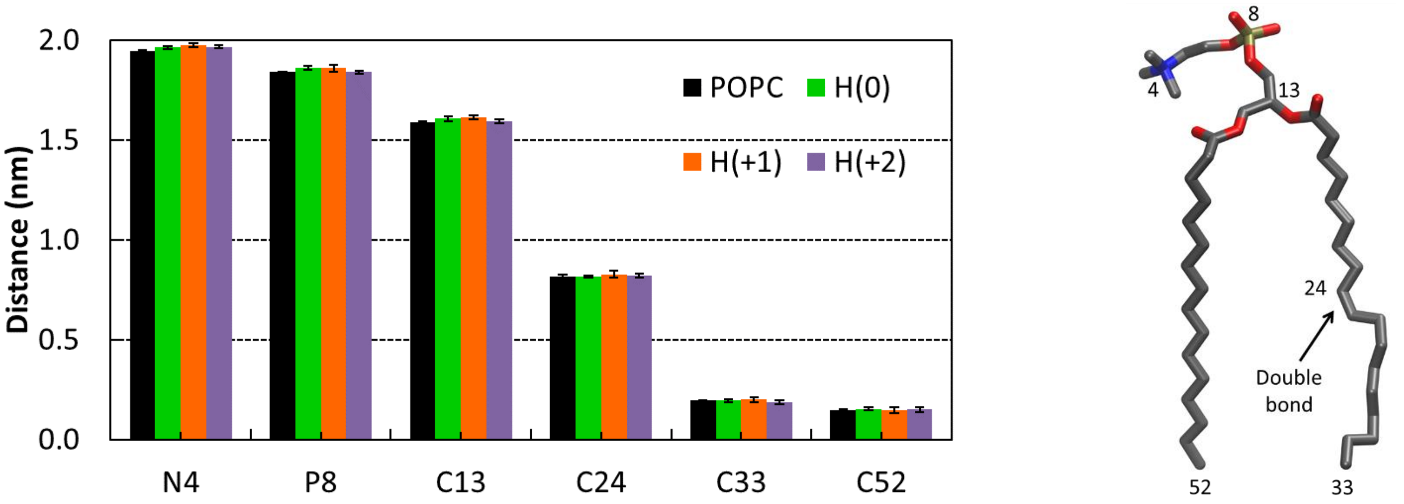

Figure S2. Average transverse distances to the bilayer center for selected atoms of POPC (numbering defined in the structure on the right), for systems without probe (POPC) and in the presence of H33342 in different ionization states (H(0), H(+1) and H(+2)).

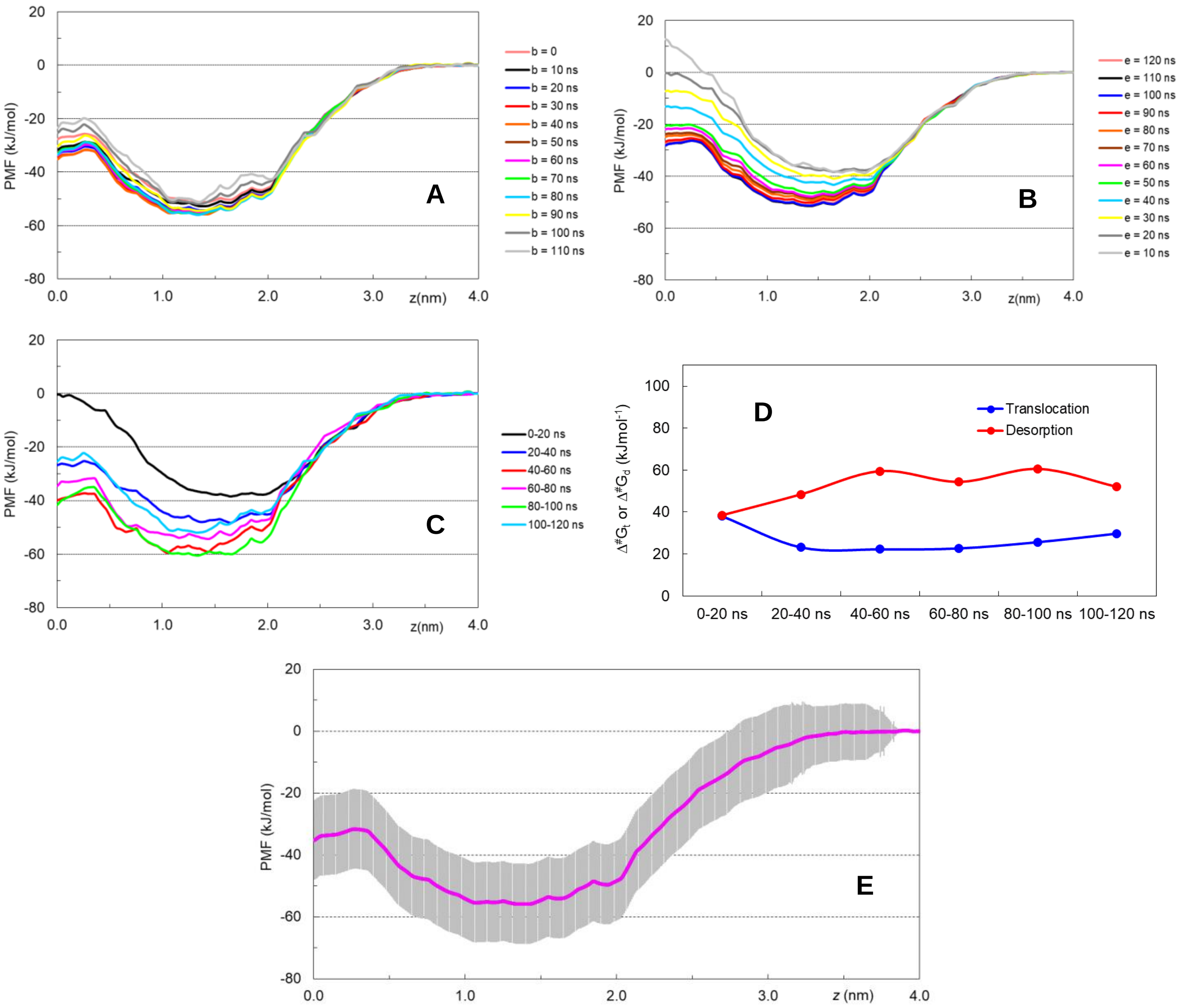

Figure S3. PMF error and convergence analysis for H(0). (A): Convergence of the PMF profiles obtained discarding initial simulation times. b = *t* denotes that times before *t* were not considered for analysis. (B): Convergence of the PMF profiles obtained discarding final simulation times. e = *t* denotes that times after *t* were not considered for analysis. (C): PMFs obtained considering different 20 ns time intervals of the sampling simulations. (D): Translocation and Desorption free energies ($\Delta^{\#}G_t$ and $\Delta^{\#}G_d$, respectively) calculated from each of the PMF curves of panel C. (E): PMF curve calculated using the last 80 ns of the 120 ns sampling runs.

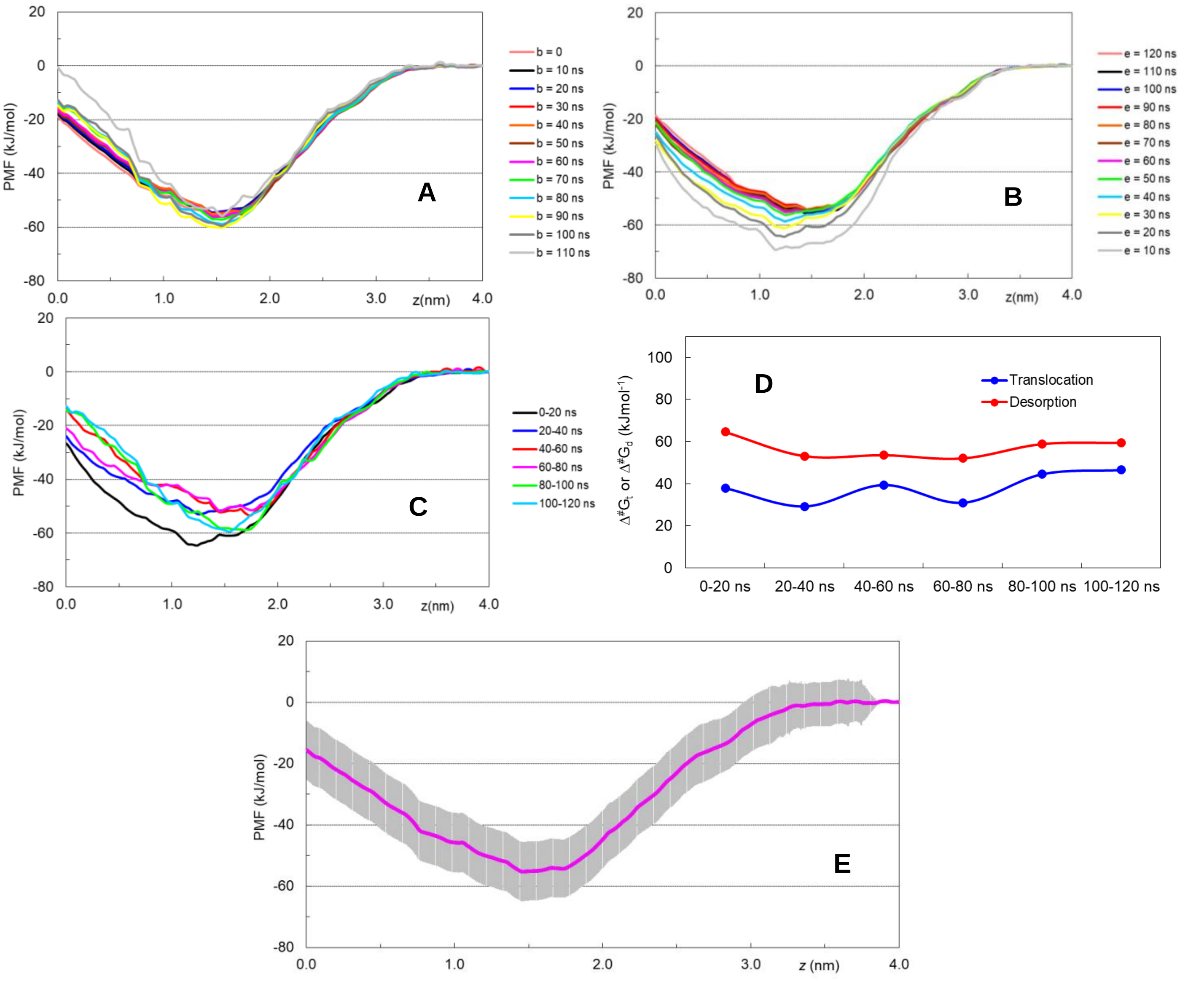

Figure S4. PMF error and convergence analysis for H(+1). Panels A-E have the same meanings as in Figure S3.

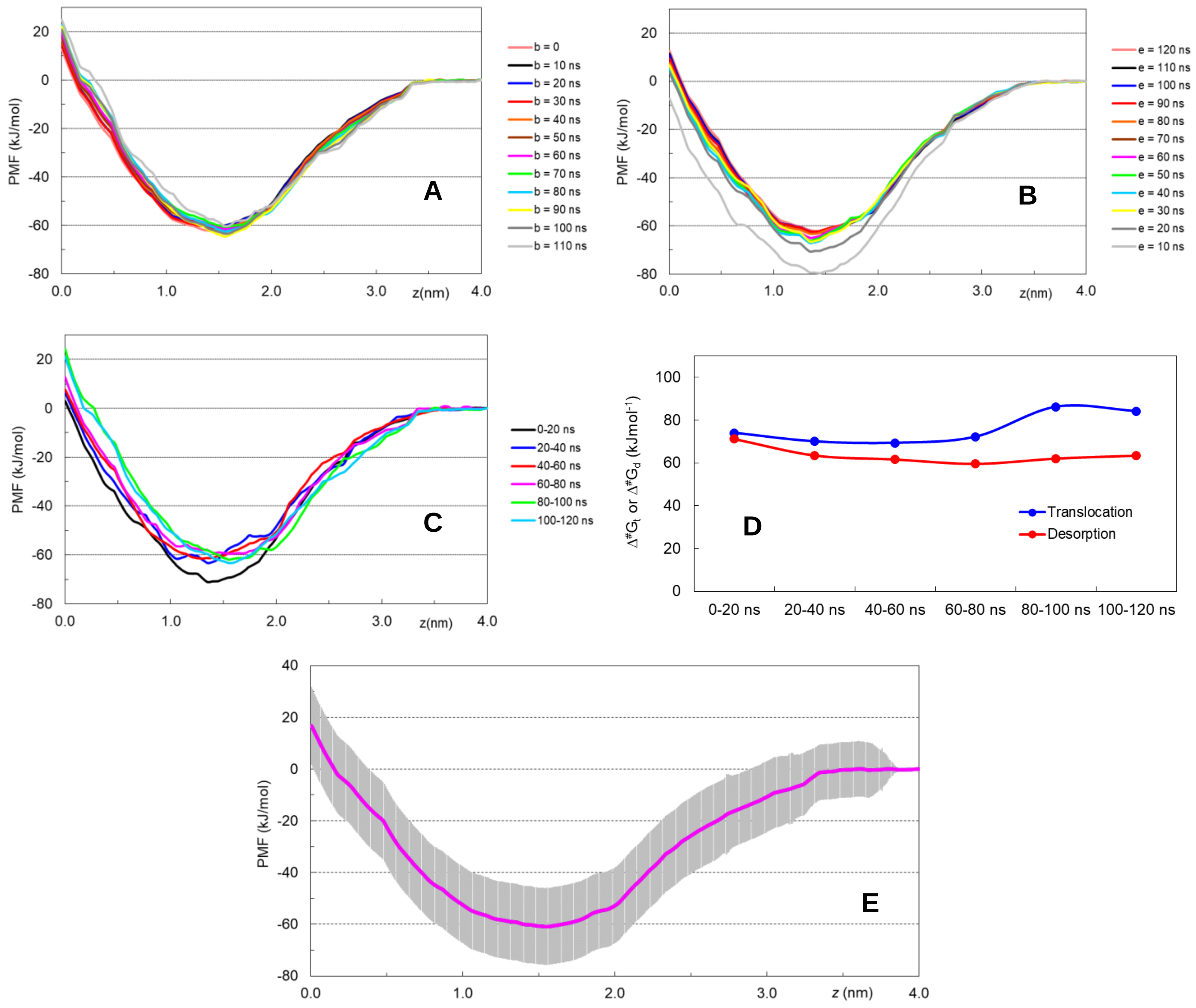

Figure S5. PMF error and convergence analysis for H(+2). Panels A-E have the same meanings as in Figure S3.
